# SIRT2 transgenic over‐expression does not impact lifespan in mice

**DOI:** 10.1111/acel.14027

**Published:** 2023-11-27

**Authors:** Lindsay E. Wu, Corrine E. Fiveash, Nicholas L. Bentley, Myung‐Jin Kang, Hemna Govindaraju, Jayne A. Barbour, Brendan P. Wilkins, Sarah E. Hancock, Romanthi Madawala, Abhijit Das, Hassina Massudi, Catherine Li, Lynn‐Jee Kim, Ashley S. A. Wong, Maria B. Marinova, Ghazal Sultani, Abhirup Das, Neil A. Youngson, David G. Le Couteur, David A. Sinclair, Nigel Turner

**Affiliations:** ^1^ School of Biomedical Sciences UNSW Sydney Kensington New South Wales Australia; ^2^ Victor Chang Cardiac Research Institute Darlinghurst New South Wales Australia; ^3^ School of Psychology UNSW Sydney Kensington New South Wales Australia; ^4^ ANZAC Medical Research Institute Concord New South Wales Australia; ^5^ Charles Perkins Centre The University of Sydney Sydney New South Wales Australia; ^6^ Department of Genetics, Blavatnik Institute Paul F. Glenn Center for Biology of Aging Research, Harvard Medical School Boston Massachusetts United States

**Keywords:** aging, aging biology, healthspan, lifespan, longevity, sirtuin 2 (SIRT2), sirtuins

## Abstract

The NAD^+^‐dependent deacylase family of sirtuin enzymes have been implicated in biological ageing, late‐life health and overall lifespan, though of these members, a role for sirtuin‐2 (SIRT2) is less clear. Transgenic overexpression of SIRT2 in the BubR1 hypomorph model of progeria can rescue many aspects of health and increase overall lifespan, due to a specific interaction between SIRT2 and BubR1 that improves the stability of this protein. It is less clear whether SIRT2 is relevant to biological ageing outside of a model where BubR1 is under‐expressed. Here, we sought to test whether SIRT2 over‐expression would impact the overall health and lifespan of mice on a nonprogeroid, wild‐type background. While we previously found that SIRT2 transgenic overexpression prolonged female fertility, here, we did not observe any additional impact on health or lifespan, which was measured in both male and female mice on standard chow diets, and in males challenged with a high‐fat diet. At the biochemical level, NMR studies revealed an increase in total levels of a number of metabolites in the brain of SIRT2‐Tg animals, pointing to a potential impact in cell composition; however, this did not translate into functional differences. Overall, we conclude that strategies to enhance SIRT2 protein levels may not lead to increased longevity.

## Sirtuin 2 (SIRT2) has been implicated in cell cycle regulation, genome stability and neurodegeneration, and its over‐expression in BubR1 hypomorph progeroid mice extends lifespan. Here, comprehensive phenotyping in male and female, chow and high fat fed mice shows no impact of SIRT2 over‐expression on healthspan or lifespan on a non‐progeroid, wild‐type background.

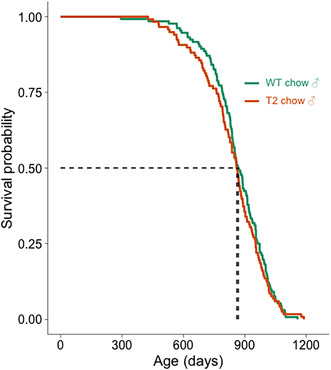

**Abbreviations**
μCT: micro computed tomography
AAV: adeno‐associated virus
ACEC: animal care and ethics committee
ANOVA: analysis of variance
ATP: adenosine triphosphate
AUC: area under the curve
BCA: bicinchoninic acid
CM: colony mice
CNS: central nervous system
EAD: exhaust air duct sample
ELISA: enzyme‐linked immunosorbent assay
fMRI: functional magnetic resonance imaging
G6PD: glucose‐6‐phosphate dehydrogenase
GABA: γ‐aminobutyric acid
GTT: glucose tolerance test
HFD: high fat diet
NAA: N‐acetyl‐aspartate
NAD^+^
: nicotinamide adenine dinucleotide
NMR: nuclear magnetic resonance
PBS: phosphate buffered saline
PCR: polymerase chain reaction
RPM: revolutions per minute
SBS: soiled bedding sample
SIRT1: sirtuin 1
SIRT2: sirtuin‐2
SIRT6: sirtuin‐6
T2: SIRT2 transgenic
Tg: transgenic
WT: wild type

## 1 INTRODUCTION

The sirtuin family of NAD^+^‐dependent deacylase family of enzymes contains seven members, playing diverse roles in epigenetic regulation, DNA repair and metabolic homeostasis. Interest in these proteins was sparked by initial findings on the role of *Sir2* in yeast replicative lifespan (Kaeberlein et al., 1999), and subsequent interest in the mammalian homologue SIRT1. This was further compounded by the identification of small molecule allosteric activators for SIRT1 (Hubbard et al., 2013), with these compounds demonstrating potential in preclinical models of disease (Massudi et al., 2016). Whole‐body transgenic over‐expression of SIRT1 impacts some aspects of late‐life health (Banks et al., 2008; Herranz et al., 2010), it does not increase overall lifespan in mice (Herranz et al., 2010), unlike the transgenic over‐expression of another nuclear sirtuin, SIRT6 (Kanfi et al., 2012; Roichman et al., 2016). This may be complicated by tissue specific effects, as unlike whole‐body overexpression, tissue specific SIRT1 overexpression in the hypothalamus results in increased overall lifespan (Satoh et al., 2013). It is currently unknown whether altered activity of other members of this family can impact mammalian ageing.

One member of this family, sirtuin‐2 (SIRT2) was previously found to regulate stability of the kinetochore attachment protein BubR1 (North et al., 2014), which has been implicated in maintaining accurate chromosome segregation during mitosis to prevent cellular senescence during ageing (Baker et al., 2011). Transgenic over‐expression of BubR1 extends lifespan (Baker et al., 2013), while its under‐expression results in the accelerated onset of age‐related pathologies and shortened overall lifespan (Baker et al., 2004, 2008; Wijshake et al., 2012). Previously, we showed that SIRT2 transgenic over‐expression (SIRT2‐Tg) in the context of BubR1 under‐expression in a hypomorph mouse strain partially rescued the lifespan of male, but not female mice (North et al., 2014). Further, we showed that this same SIRT2‐Tg allele on a non‐progeroid wild‐type (WT) C57BL6 background could delay reproductive ageing in mice, with an extended period of functional fertility (Bertoldo et al., 2020). While members of the sirtuin family have been classically studied as NAD^+^‐dependent deacetylase enzymes, SIRT2 is also capable of removing other acyl modifications on lysine residues, including lactylation (Zu et al., 2022), crotonylation (Bao et al., 2014), myristoylation (Feldman et al., 2015) and benzoylation (Huang et al., 2018). Well‐studied substrates for SIRT2 include tubulin (North et al., 2003), histones, glucose‐6phospohate dehydrogenase (G6PD) (Wang et al., 2014; Xu et al., 2016), Foxo1, Foxo3a, p65 (Rothgiesser et al., 2010), IDH1 (Wang et al., 2020), APC^CDH1^ (Kim et al., 2011) and others. SIRT2 has been proposed as a therapeutic target in neurodegenerative disease, though a number of these findings are contradictory (Chen et al., 2021; Li & Wu, 2021). For example, SIRT2 inhibitors can provide neuroprotection against models of Huntington's disease (Luthi‐Carter et al., 2010), Alzheimer's disease (Bai et al., 2022) and Parkinson's disease (de Oliveira et al., 2017; Outeiro et al., 2007); however, SIRT2 deletion impairs axonal energy metabolism, resulting in locomotor disability (Fourcade et al., 2017), with SIRT2 also playing a putative role in reducing neuroinflammation (Chen et al., 2021). Similarly, there is evidence for SIRT2 as both a suppressor and promoter of tumour growth (Zhang et al., 2020), though these roles are likely to be context dependent.

Given this previous work, the aim of this investigation was to establish whether SIRT2 could impact overall lifespan on a non‐progeroid background in mice. Here, we characterized aspects of metabolism, development, motor coordination, mitochondrial function, bone health, fertility and overall lifespan in a previously described mouse strain over‐expressing SIRT2 (Bertoldo et al., 2020; North et al., 2014). While SIRT2 over‐expression had impacts on levels of certain metabolites in the brain, we found no impact of SIRT2 overexpression any aspect of overall health or lifespan, suggesting that levels of this protein are not relevant to functional biological ageing and lifespan under standard, non‐progeroid conditions.

## 2 RESULTS

**FIGURE 1:**
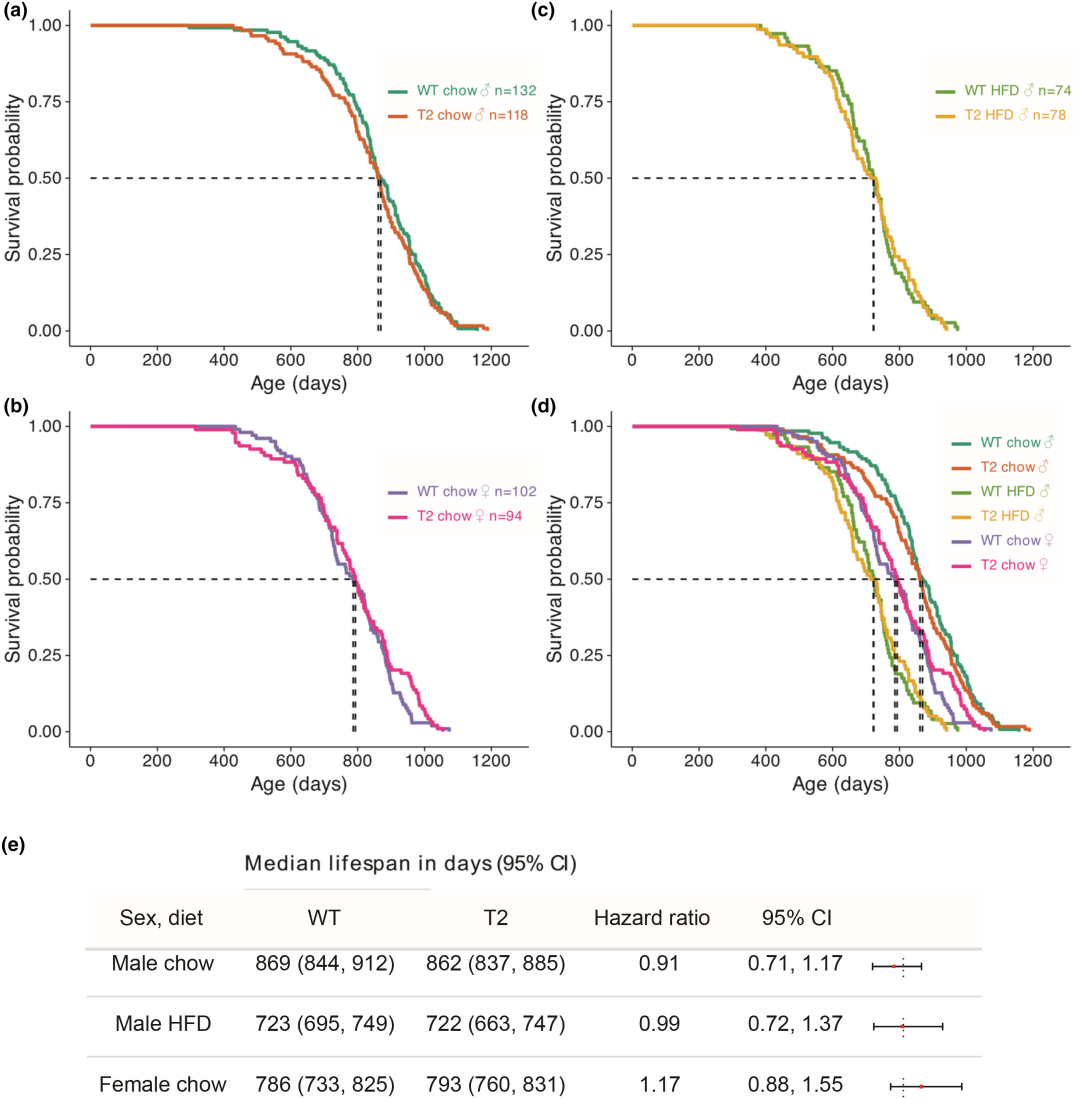
SIRT2 does not impact overall lifespan. Kaplan–Meier survival curves for SIRT2‐Tg mice separated into ((a) male and b) female mice on a standard chow diet, and (c) males fed a high‐fat diet (HFD), with all data overlaid and summarized in panel (d). Dotted lines indicate median survival, also shown in (e) with hazard ratios and 95% confidence intervals.

Using a cohort of animals globally over‐expressing SIRT2 under the CAGGS promoter (North et al., 2014), we sought to test the impact of artificially elevating SIRT2 protein on health and lifespan in mice. Given that we previously identified an impact of SIRT2 overexpression on male, but not female mice, we used both males and females for this study. Further, we also tested the impact of high‐fat diet (HFD)‐induced obesity in male mice, as this intervention can accelerate age‐related pathologies and shorten overall lifespan. This experiment was powered to detect a 10% difference in median lifespan, with power (1‐*β*) of 0.8, and *α*‐error probability of 0.05. To measure overall lifespan, animals were maintained within the UNSW animal facility from 8 weeks of age until they reached humane endpoints requiring euthanasia (described in further detail in Methods) as required by the UNSW animal care and ethics committee (ACEC), or when found dead. The age of death was used to construct Kaplan–Meier survival curves (Figure 1), which were analysed for differences in survival using the Cox proportional hazards test. SIRT2 over‐expression had no effect on lifespan in chow‐fed male mice, with a median survival of 869 days in WT animals compared to 862 days in SIRT2Tg mice (Figure 1a). Similarly, there was no difference in chow‐fed female mice, with a median lifespan of 723 versus 722 days in WT and SIRT2‐Tg animals, respectively (Figure 1b).

Finally, there was no impact SIRT2 over‐expression in male mice fed a high‐fat diet (HFD), with median lifespans of 786 versus 793 days (Figure 1c). As expected, animals fed a HFD had a shorter overall lifespan (Figure 1d), living around 140 days shorter than their chow‐fed littermates (Figure 1e), which is consistent with a well‐described role for the lipids component of overall macronutrient intake as a determinant of lifespan (Solon‐Biet et al., 2014). One surprising observation was that females in this cohort had shorter lifespans than males (Figure 1d,e), which is contrary to the consistent trend across evolution for females to live longer than males (Xirocostas et al., 2020); however; this appears to be a feature of the C57BL6 mouse strain that has been observed previously (Turturro et al., 1999).

**FIGURE 2:**
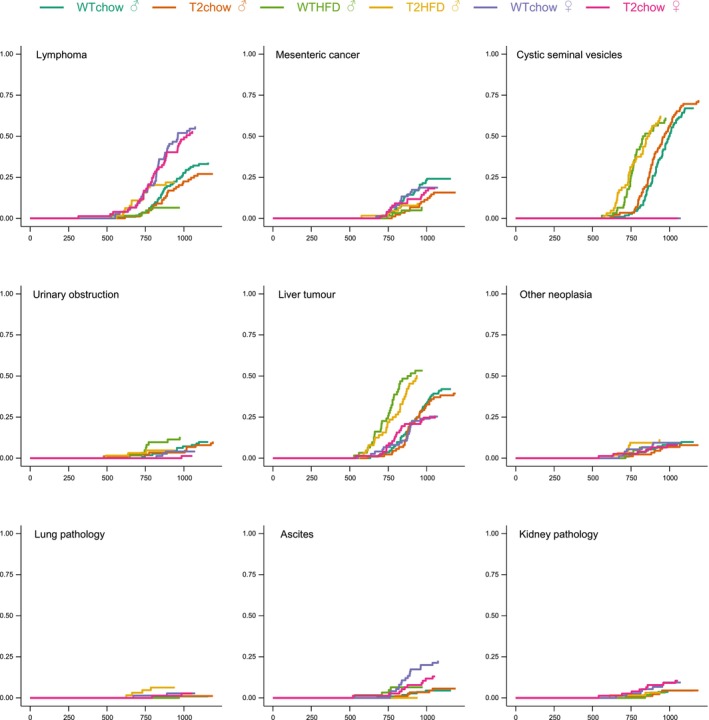
Cumulative incidence of post‐mortem pathologies in SIRT2‐Tg and WT littermate control mice. Where possible, all animals in the lifespan study were subject to post‐mortem necropsy to determine the presence of tumours, with the overall incidence for each group shown—where necropsy was not possible, animals were excluded from this analysis.

Due to animal ethics requirements, a condition of our study was that animals would be euthanased once they had reached a composite score of clinical endpoints (described in Methods), rather than allowing natural death as an endpoint. We therefore plotted the most immediate symptom that was noted for requiring euthanasia for each group (Figure 2a). Although there were sex‐ and diet‐specific impacts on reasons for euthanasia, these data revealed no impact from SIRT2 overexpression. Where possible, animals that were subject to gross necropsy following euthanasia or being found dead, with the cumulative incidence of the most common pathologies also shown (Figure 2b). While the most common macroscopic observation of neoplasia was liver tumours, these were likely secondary to hematological malignancies, which are common in aged mice. In line with this, evidence of lymphoma (enlarged spleens, lymph nodes) were common, with an increased incidence in females (Figure 2b).

**FIGURE 3:**
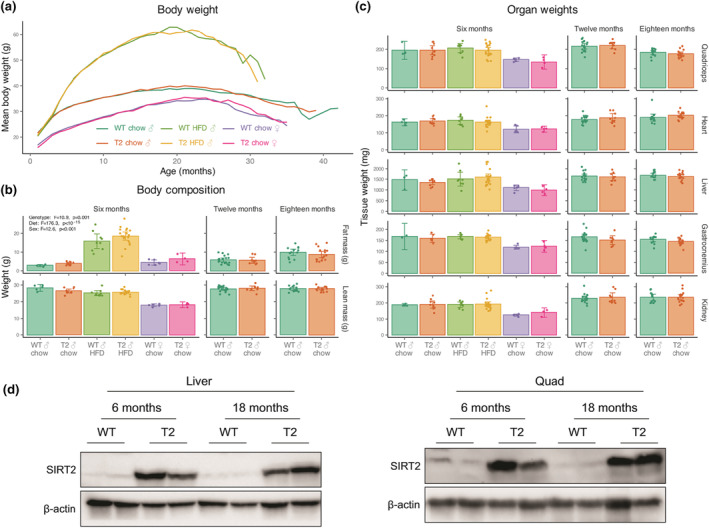
(a) Body weight, (b) body composition determined by quantitative MRI and (c) organ weights in male and female SIRT2‐Tg animals and their WT littermates maintained on a standard lab diet (chow) or high‐fat diet (HFD) at 6 months of age, followed by recordings for chow fed males at 12 and 18 months of age. (d) SIRT2 protein overexpression in liver and quadricep tissue from 6 and 18‐month old chow‐fed males, demonstrating over‐expression is maintained with age. Data analysed by multifactorial ANOVA, results annotated only where significant. Each dot represents data from a separate animal, error bars are 95% confidence intervals.

**FIGURE 4:**
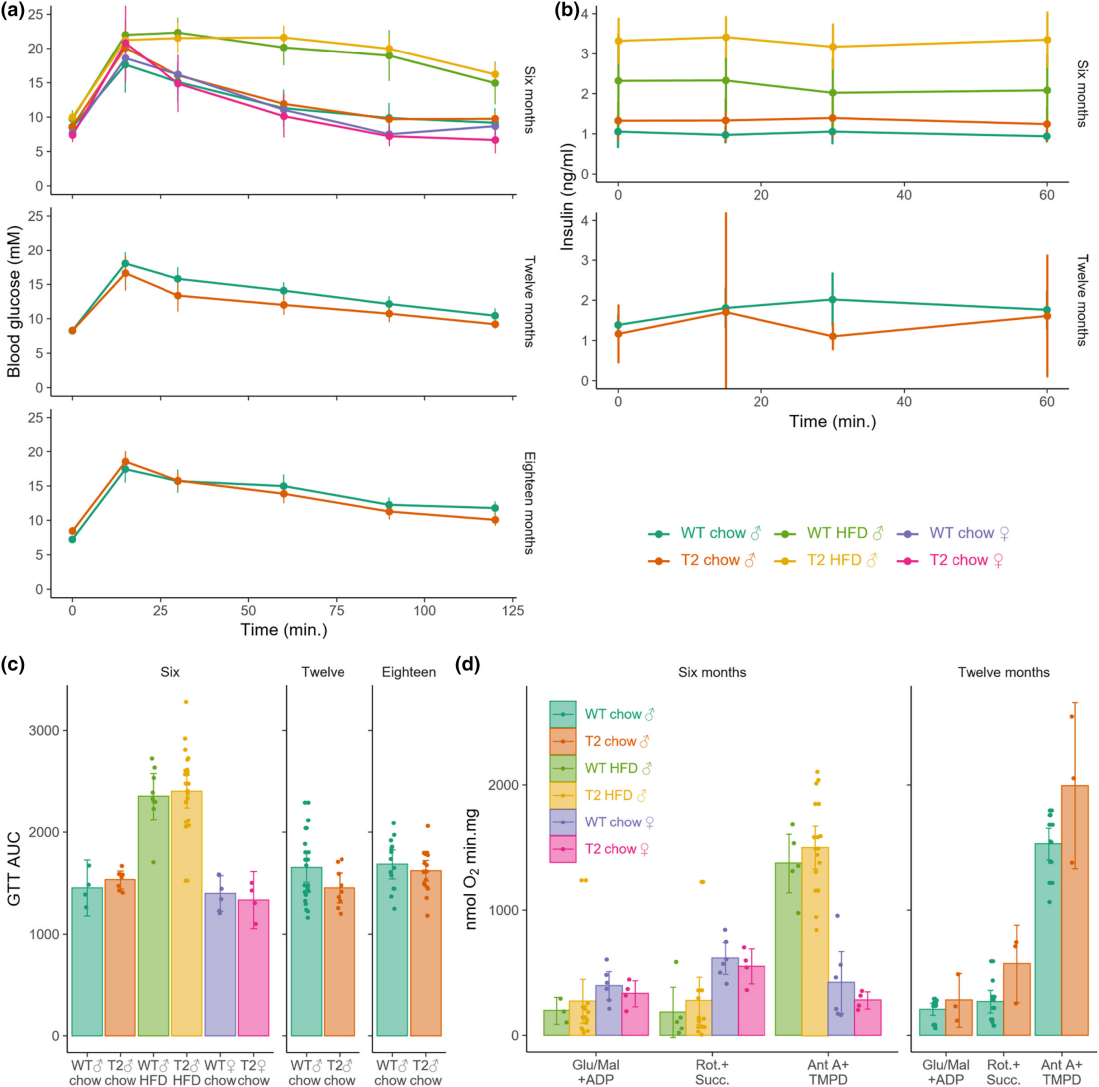
Metabolic parameters of SIRT2‐Tg mice. (a) Glucose tolerance tests (GTT) were conducted at indicated age groups, with (b) circulating insulin during the GTT. Data were summarized into (c) area under the curve (AUC) measurements. (d) Mitochondrial function was assessed from the livers using a Clarke‐type electrode to measure State III respiration of Complex I, II and IV. Error bars are SD, each dot represents observation from a separate animal.

**FIGURE 5:**
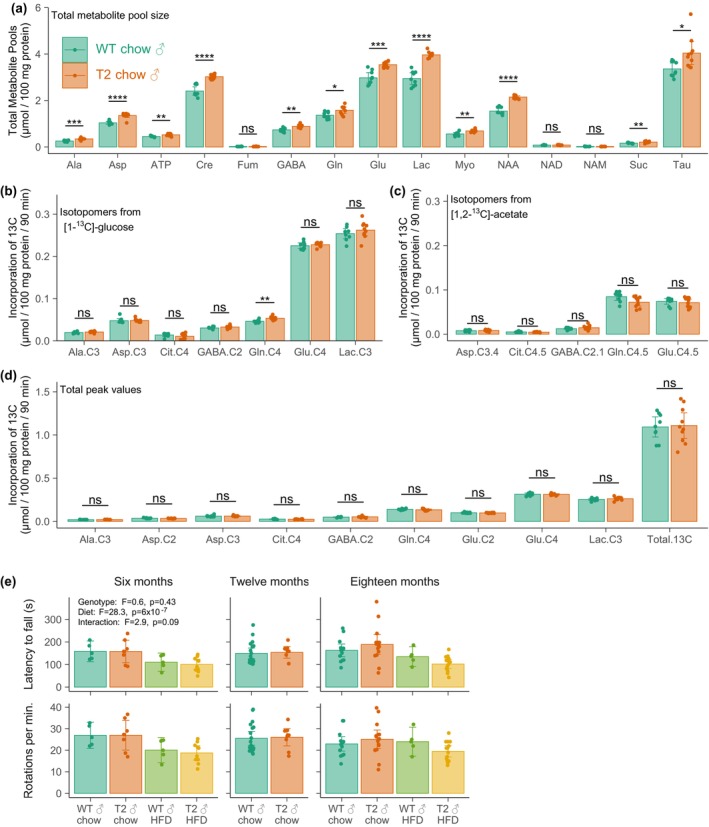
Brain metabolic flux in SIRT2‐Tg mice. Animals were administered with [1‐^13^C]‐glucose and [1,2‐^13^C]‐acetate 90 min prior to euthanasia, with brain cortex samples subject to ^1^H and ^13^C NMR to measure (a) total metabolite levels, rates of incorporation into metabolite pools from (b) [1^13^C]‐glucose and (c) [1,2‐^13^C]‐acetate, with (d) total ^13^C incorporation shown. Any differences in brain metabolite levels did not translate into (e) differences in performance of animals on an accelerating rotarod. Data analysed in (a–d) by two‐tailed *t*‐test with significance adjusted using a Bonferroni correction for multiple comparisons, in (E) by two‐way ANOVA. *p < 0.05, **p < 0.01, ***p < 0.001, ****p < 0.0001.

**FIGURE 6:**
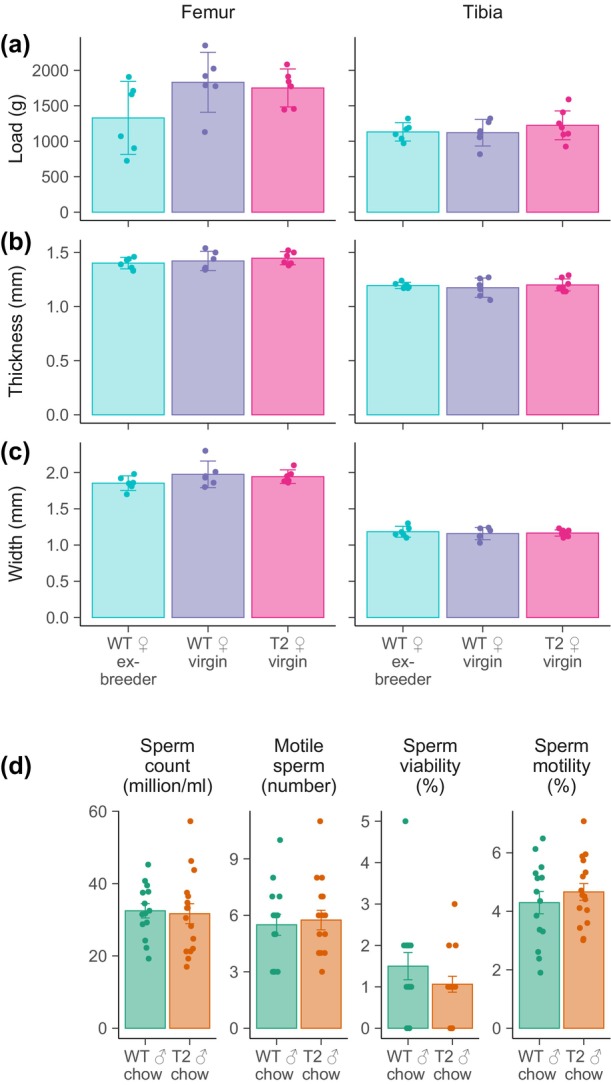
Health parameters linked to reproductive ageing. (a–c) Bone health in reproductively aged (14‐month‐old) female SIRT2‐Tg mice. Animals were euthanased at 14 months of age, and bones from hindlimbs collected for (a) mechanical testing to establish the load required to break femur or tibia bones, and to measure (b) bone thickness (anterior–posterior) and (c) width (lateral–medial), as indicated. (d) Sperm count, motility and viability in 18‐ to 19‐month‐old chow fed WT and SIRT2‐Tg male littermates.

In parallel with this study of overall lifespan, a separate cohort of animals were subject to serial measures of healthspan at 6, 12 and 18 months of age, including overall body weight (Figure 3a), body composition (Figure 3b), tissue weight (Figure 3c), glucose homeostasis (Figure 4a–c), mitochondrial function (Figure 4d), brain metabolomics (Figure 5), motor coordination (Figure 5e), bone health (Figure 6) and sperm quality (Figure 6d). These measures were taken in a parallel cohort of animals that was separate to those used in the overall lifespan study, to avoid interference from tests that could impact overall lifespan. As expected, body weight was increased in males compare to females, and were almost doubled in those fed a high‐fat diet compared to those maintained on a chow diet, although there was no change in weight due to genotype (Figure 3a). There was, however, a slight impact of genotype on body composition (Figure 3b), with SIRT2‐Tg mice having slightly greater adiposity at younger ages, which came at the cost of decreased lean mass (Figure 3b). This may be related to the regulation of the pentose phosphate pathway enzyme glucose‐6‐phosphate dehydrogenase (G6PD) by SIRT2 (Wang et al., 2014; Wu & Sinclair, 2014), which may be important to controlling the rate of de novo lipogenesis in certain situations (Xu et al., 2016). Cohorts of animals were sacrificed at 6, 12, and 18 months for tissue collection, with no impact of SIRT2 over‐expression on organ weight (Figure 3c). To check that the lack of impact from SIRT2 overexpression on lifespan (Figure 1) was not due to silencing of the SIRT2 transgene, tissues from 6‐ and 18‐month‐old chow fed males were subject to western blotting for SIRT2 protein levels (Figure 3d). These blots confirmed no loss of SIRT2 transgene overexpression, which was maintained at >10‐fold higher than those of wild‐type littermates.

Next, we sought to test whether SIRT2 overexpression had any impact on glucose homeostasis and metabolism, as overexpression of this enzyme can promote hepatic glucose uptake (Watanabe et al., 2018) and overall glucose homeostasis (Lemos et al., 2017), while its deletion can impair insulin secretion and glucose homeostasis (Zhou et al., 2021). As expected, HFD feeding impaired glucose clearance during a glucose tolerance test (GTT) (Figure 4a); however, there was no impact of SIRT2 over‐expression in either sex or diet. Although there was a trend towards increased insulin levels during the GTT (Figure 4b) that could be expected based on previous work (Zhou et al., 2021), this was within the range of error and not statistically significant. In previous work, improvements in glucose homeostasis with SIRT2 overexpression were accompanied by protection against mitochondrial dysfunction in the liver (Lemos et al., 2017), with others demonstrating a role for SIRT2 in mitochondrial dynamics following its knockdown or deletion (Cha et al., 2021; Liu et al., 2017). We isolated mitochondria from the livers of our SIRT2‐Tg animals and their WT littermates and used a Clarke‐type electrode to measure oxygen consumption in the presence of substrates and inhibitor combinations that allowed us to quantify Complex I, II and IV of the mitochondrial electron transport chain (Figure 4d). In contrast to previous work, we observed no genotype effect within any age, diet or sex comparisons.

SIRT2 has been studied extensively for its potential role in neurogenerative diseases, including through regulating aspects of brain metabolism (Chamberlain et al., 2021; Luthi‐Carter et al., 2010; Ma et al., 2022). To address this, we traced the metabolism of ^13^C isotope labelled glucose and acetate into the cerebral cortex of SIRT2‐Tg and WT littermates as previously described (Nilsen et al., 2013), measuring both ^13^C label incorporation and total metabolite levels using ^1^H and ^13^C NMR spectroscopy (Figure 5). ^1^H‐NMR for total metabolite levels revealed increases in alanine (Ala), aspartate (Asp), ATP, creatine, GABA, glutamine (Glu), lactate (Lac), *myo*‐inositol (Myo) and *N*‐acetyl‐aspartate (NAA) in SIRT2‐Tg animals compared to their WT littermates (Figure 5a). NAA has been used as a marker of mitochondrial integrity in neurons (Bates et al., 1996) due to its exclusive production in neurons (Rigotti et al., 2007; Tallan et al., 1956; Urenjak et al., 1992), and its elevated levels in SIRT2‐Tg animals could be indicative of neuronal health. Similarly, we observed an increase in GABA levels which decline with ageing, although this trend may reflect anatomical changes rather than total levels (Maes et al., 2018). Together, the increase in these and other metabolites including ATP, creatine and amino acids could indicate an impact of SIRT2 overexpression on neuronal function. When measuring ^13^C label incorporation from either [1‐^13^C]‐glucose (Figure 5b) or [1,2‐^13^C]‐acetate (Figure 5c), SIRT2 overexpression did not impact label incorporation with the exception in a reduction in Gln‐C4,5 labelling, (Figure 5c), which is thought to reflect glutamine production from acetate in astrocytes and oligodendrocytes due to the largely selective expression of glutamine synthetase in those cells (Norenberg & Martinez‐Hernandez, 1979; Rowlands et al., 2017; Xin et al., 2019). Although astrocyte‐derived glutamine can be delivered to neurons for its incorporation into GABA production, despite the reduction in Gln‐4,5 labelling, GABA levels were instead elevated in SIRT2Tg mice—likely instead reflecting the role of neuronal mitochondrial activity in GABA production through a glutamate intermediate (Rowlands et al., 2017), which was also consistent with increased glutamate levels (Figure 5a). Despite this, we did not observe a change in ^13^C label incorporation into GABA labelling, nor did we observe changes in C4 or C4,5‐Glu labelling, which would indicate production from glucose and acetate, respectively. This could suggest that any difference in the rate of incorporation into these labels is beyond the timescale of our labelling experiment, or alternatively, that the change in GABA levels is due to production via the polyamine pathway. Given the role of GABA in motor coordination (Stagg et al., 2011) and preclinical findings regarding the role of SIRT2 in locomotor function in ageing and disease (Esteves et al., 2018; Godena et al., 2014; Wang et al., 2015), animals were also subjected to the accelerating rotarod test (Figure 5e) to assess their motor coordination in ageing. No impact of SIRT2 overexpression observed in any age group, in line with other findings that show no impact of SIRT2 overexpression, deletion or chemical inhibition on rotarod performance (Bobrowska et al., 2012; Erburu et al., 2017; Sola‐Sevilla et al., 2021).

Our previous work revealed a potential role for SIRT2 in female fertility, with improved oocyte quality, ovulation rates and pregnancies during ageing in SIRT2 transgenics, with SIRT2‐Tg mice still able to maintain pregnancies at the late reproductive of age 14–16 months of age (Bertoldo et al., 2020). Female reproductive ageing is closely linked to other aspects of health due to a decline in oestrogen secretion which occurs once the ovarian reserve has been exhausted. This decline in oestrogen has its most noticeable impacts on bone, which depends on oestrogen secretion to maintain an appropriate balance between osteoclast and osteoblast activity. To test whether these previously observed changes in fertility translated to altered bone health, we sacrificed a cohort of female animals at 14 months of age to assess their bone health (Figure 6). Given the impacts of pregnancy on late‐life endocrine function, we used a separate cohort of age‐matched ex‐breeder WT animals as a comparison. Following tissue cleaning, hindlimb femurs and tibias were subject to mechanical testing and μ‐CT imaging, which revealed no changes in overall bone strength between WT and SIRT2‐Tg animals (Figure 6). Although no change was observed, future work should aim to assess oestrogen levels in SIRT2‐Tg animals following blood collection from animals at an identical phase of oestrous.

In light of our previous findings around the extended female fertility of SIRT2‐Tg animals, we next sought to test whether SIRT2 overexpression would similarly impact male fertility and sperm function. As with oocytes, sperm are also critically dependent on levels of the kinetochore attachment protein BubR1, whose levels decline in the testis with age (Baker et al., 2004), and might benefit from SIRT2 overexpression due to its stabilization of this protein (North et al., 2014). In line with other results from this study, there was no difference in sperm counts, motility or viability in SIRT2‐Tg animals (Figure 6d).

## 3 DISCUSSION

Interest in the biology of the sirtuins has been driven by potential roles for these proteins in biological ageing, with a role for these NAD^+^‐dependent enzymes potentially acting as an explanation for the impact of declining NAD^+^ levels during old age, and the beneficial effects of reversing this decline during ageing and other pathologies through treatment with exogenous NAD^+^ metabolites (Aflatounian et al., 2022; Das et al., 2018; Rajman et al., 2018; Wu & Sinclair, 2016; Zhang et al., 2016). Previous work showed that SIRT2 overexpression could drastically rescue overall lifespan in a progeria mouse model (North et al., 2014), and we also showed that this same strain of SIRT2‐Tg mice had improved oocyte quality and female fertility later in life (Bertoldo et al., 2020). Further, polymorphisms in this gene are associated with human longevity (Crocco et al., 2016), providing further interest in the role of this protein in ageing. Here, we show that under standard laboratory conditions, SIRT2 levels are not rate‐limiting to lifespan and late‐life health in wild‐type mice, with no impact from the overexpression of this protein on overall lifespan (Figure 1). The discordance between the present findings and previous work on a background of the BubR1 hypomorph progeria model likely relates to the relevance of the mechanistic role for SIRT2 in the stabilization of BubR1 protein (North et al., 2014). This interaction is likely relevant in models where BubR1 levels are artificially reduced to the point of being rate limiting to lifespan; however, may not be relevant to biological ageing under normal circumstances. While BubR1 over‐expression can increase overall lifespan (Baker et al., 2013), it is likely that any stabilization of BubR1 by SIRT2 under normal ageing is not enough to increase BubR1 levels to the point of having an impact on lifespan. In addition to lifespan, we show no impact of SIRT2 overexpression on age‐related parameters of health, including tumour incidence (Figure 2), glucose homeostasis (Figure 4a), mitochondrial function (Figure 4d), motor coordination (Figure 5e), bone health (Figure 6) or sperm function (Figure 6d). These findings are in line with recent work which found no impact from expression of the SIRT2.3 isoform of SIRT2 in the CNS on senescence (Sola‐Sevilla et al., 2021).

In this study, we did not observe any impact of SIRT2 over‐expression on whole‐body glucose homeostasis (Figure 4a–c). Previous work has implicated a role for SIRT2 in glucose homeostasis, through its actions on hepatic glucose output (Ren et al., 2021; Watanabe et al., 2018), glucose stimulated insulin secretion (Zhou et al., 2021) and in vitro models of insulin sensitive tissues (Arora & Dey, 2014; He et al., 2020; Lemos et al., 2017; F. Wang & Tong, 2009). These previous studies have included AAV‐mediated SIRT2 over‐expression in the liver (Ren et al., 2021; Watanabe et al., 2018), against a background of metabolic dysfunction similarly achieved using high fat feeding (Ren et al., 2021), resulting in improved glucose tolerance due to reduced hepatic glucose output. One possible reason for the discordance between those results and this study (Figure 3a–c) could be the opposing action of SIRT2 in other insulin‐sensitive tissues, yet other work on SIRT2 in these tissues has suggested the opposite. For example, SIRT2 is supposedly upregulated with fasting and represses adipogenesis, however these results were obtained from in vitro studies of 3T3‐L1 adipocytes and not from in vivo studies (Wang & Tong, 2009). Overexpression of SIRT2 in C2C12 cells (Arora & Dey, 2014) has very little impact in insulin sensitivity in C2C12 cells; however, these are a very poor model for insulin stimulated glucose uptake and these results are further complicated by the use of the fluorescent glucose analogue NBDG, which is fraught with artefacts (Hamilton et al., 2021). Co‐culture of SIRT2 overexpressing macrophages with aged WT adipose tissue reduced insulin resistance, but the only read out for this is Akt phosphorylation (He et al., 2020) which does not reflect actual insulin action, as mediated by GLUT4 translocation (Hoehn et al., 2008). Finally, SIRT2 knockouts have impaired glucose tolerance and insulin secretion (Zhou et al., 2021); however, caution should be warranted in extrapolating this finding to assume that over‐expressing SIRT2 will have the opposite effect of its deletion.

Although SIRT2 has been proposed to play a role in various preclinical models of disease, these models have been induced by genetic manipulation, chemical treatment or injury, rather than spontaneous causes of disease that occur without intervention as a result of ageing. For example, SIRT2 has been proposed to play a role in Alzheimer's disease and Parkinson's disease, two common pathologies which spontaneously occur with ageing, and are modelled in rodents using transgenic or chemical insults (de Oliveira et al., 2017; Esteves et al., 2018; Godena et al., 2014; Outeiro et al., 2007; Yan et al., 2022), leading to medicinal chemistry efforts to develop small molecule inhibitors against this protein (Hong et al., 2019; Nielsen et al., 2021; Outeiro et al., 2007). Despite this, we observed no change in rotarod performance (Figure 5e). Similarly, SIRT2 maintains genome integrity in the context of carcinogen‐induced cancer models, limiting tumour growth (Kim et al., 2011). Again, we observed no change in the overall incidence of spontaneous tumours in SIRT2‐Tg mice, including no change in the frequency of liver tumours (Figure 2). Overall, we found no impact of SIRT2 overexpression on the spectrum of mortality in mice.

One surprising finding from this study was the impact of SIRT2 overexpression on the levels of several brain metabolites, notably an elevation in NAA (Figure 5a), which is a marker of neuronal content. This was complemented by a reduction in [1,2‐^13^C]‐acetate incorporation into glutamine C4,5 labelling (Figure 5c), which reflects the metabolism of acetate in astrocytes and oligodendrocytes (Amaral et al., 2016). Given that SIRT2 is highly expressed in neurons (Jayasena et al., 2016), these data could reflect a change in the overall cell composition of the brain. These NMR measurements were from homogenized brain samples, and future work should aim to test the idea this concept of altered cell composition through segmentation of fMRI imaging (Tal et al., 2012). Alternatively, different brain regions could be dissected and analysed separately for NMR; however, we did not do this here due to the NMR acquisition time required for very small samples. Recently, SIRT2 overexpression in neurons was found to elevate ATP production (Chamberlain et al., 2021), which is in line with our findings (Figure 5a). Although we conducted assays of mitochondrial respiratory capacity (Figure 4d) and observed no change, these were from liver, rather than brain samples.

Potential study limitations for this work could include that our lifespan measurements did not reflect the natural age of death, as we euthanased animals once they reached a humane endpoint due to our animal ethics requirements. We believe that these endpoints would only have shortened overall lifespan measurements by a small number of days, as the clinical checklist requiring euthanasia typically indicated conditions that would have shortly led to natural death. Further, the median survival of animals in this study was in line with previous benchmarks for survival of this strain, with median survival of males in this study at 28.5 months, similar to the approximate median lifespan of 28 months that has been reported in the C57BL6 strain by others (Graber et al., 2015). Another limitation of this work is that although we observed changes in metabolite levels and isotope labelling that could suggest changes in brain cell type composition, our functional measures of neuronal composition were limited to a single assay for motor coordination (Figure 5e), whereas direct measures of memory may have been more appropriate. It would also have been ideal to study the impact of HFD feeding in both sexes, rather than just males, as there is strong evidence for sexual dimorphism in the response to altered dietary intake. Further, our analyses of tissues at later timepoints were restricted to males only, as the females that had been set aside at the time for this were instead used for separate studies of female fertility (Bertoldo et al., 2020). Despite these limitations, this work provides convincing evidence that increasing SIRT2 protein levels does not impact overall lifespan in mice.

## 4 METHODS

### 4.1 Animals

#### 4.1.1 Ethics

All experiments were approved by the UNSW animal care and ethics committee (ACEC) under protocol numbers 16/1A, 15/134A and 13/134B. The UNSW ACEC operates under guidelines from the National Health and Medical Research Council (NHMRC) of Australia.

#### 4.1.2 SIRT‐Tg animals

Global SIRT2 transgenic over‐expression was driven by the CAGGS promoter, using a previously described strain of animals (North et al., 2014). Animals were maintained on the C57BL6/J^Ausb^ background, which are a colony of C57BL6/J mice imported from Jackson Laboratories in 2011 and refreshed in 2014. Animals were bred at the Australian BioResources (ABR) facility in Moss Vale, NSW, Australia and transported to UNSW at 6–8 weeks of age. The colony was maintained by breeding SIRT2‐Tg heterozygous males with WT females, to avoid the chance of maternal epigenetic imprinting effects. All comparisons were between WT and SIRT2‐Tg heterozygous littermates, with the expected Mendelian ratio of genotypes observed in litters. Prior to shipping to UNSW, animals were ear‐clipped for identification, and ear clips retained for DNA extraction and genotyping at the Australian Cancer Research Foundation (ACRF)/Garvan Molecular Genetics (GMG) core facility. Genotyping information was maintained as an electronic record independently of personnel conducting animal lifespan experiments to ensure researchers remained blinded to animal genotypes.

#### 4.1.3 Genotyping

Animals were genotyped using DNA extracted from ear clips, using a PCR based protocol. The PCR reaction contained three primers: A1 (GCACAGCATTGCGGACATGC) and B (CCCTCCATGTGTGACCAAGG) targeted to the ColA1 locus, C1 (GCAGAAGCGCGGCCGTCTGG) targeted to the FRT region of the transgene (North et al., 2014). WT animals yielded a single amplicon at 331 bp, heterozygous SIRT2‐Tg animals yielded both the WT amplicon and a second amplicon at 551 bp. PCR conditions were denaturation at 95°C for 30 s, annealing at 65°C for 30 s, extension at 72°C for 60 s, repeated for 30 cycles.

#### 4.1.4 Housing

Once delivered to UNSW, animals were maintained in individually ventilated cages at a density of two to five animals per cage. Once group housed, mal mice were not re‐housed in cages with different animals, to avoid the risk of fighting. The UNSW animal facility is maintained at a temperature of 22 +/−1°C, 80% relative humidity and on a 12:12 h light/dark cycle (lights on at 0700, off at 1900 h). Animals had ad libitum access to food and water, and cages were changed on a weekly basis. Chow diet was a naturally sourced composition from Gordon's Specialty Feeds in Yanderra, NSW Australia and was 8% calories from fat, 21% from protein, and 71% from carbohydrate, with an energy density of 2.6 kcal/g, and was sterilized by irradiation. High‐fat diet (HFD) was prepared in‐house, and was based on D12451 from Research Diets, New Brunswick, NJ, United States. This diet contained 45% calories from fat (beef lard), 20% from protein and 35% from carbohydrate, at an energy density of 4.7 kcal/g. HFD was replaced weekly to avoid feed becoming rancid at room temperature, as it included ingredients (beef lard) normally stored under refrigerated conditions. Animals were monitored (body weight and visual inspection of general condition) on a weekly basis until 24 months of age, following which animals were inspected twice weekly. As detailed in the lifespan section below, a scoring sheet was used to record adverse pathologies, with a score over 2 resulting in daily inspection.

#### 4.1.5 Pathogen status

Both the breeding facility (ABR Moss Vale) and holding facility (UNSW BRC) are maintained pathogen‐free, with regular surveillance for pathogens listed in Table 1.

**TABLE 1:** Pathogens subject to regular surveillance at ABR Moss Vale and UNSW BRC animal facilities to maintain pathogen‐free status.

Pathogen	Sample	Test method
Viruses		
Mouse hepatitis virus	SBS/EAD	Serology/PCR
Mouse parvovirus	SBS/EAD	Serology/PCR
Minute virus of mice	SBS/EAD	Serology/PCR
Mouse rotavirus	SBS/EAD	Serology/PCR
Theiler's encephalomyelitis virus	SBS/EAD	Serology/PCR
Mouse norovirus	SBS/EAD	Serology/PCR
Pneumonia virus of mice	SBS/EAD	Serology/PCR
Adenovirus type 1&2	SBS	Serology
Ectromelia	SBS	Serology
Hantavirus	SBS	Serology
LCMV	SBS	Serology
Murine cytomegalovirus	SBS	Serology
Polyoma virus	SBS	Serology
Reovirus type 3	SBS	Serology
Sendai virus	SBS	Serology
K Virus	SBS	Serology
Mouse kidney parvovirus	SBS/CM	PCR
Bacteria		
*Mycoplasma pulmonis*	SBS/EAD	Serology/PCR
*Helicobacter* spp.	SBS/CM	PCR
*Citrobacter rodentium*	SBS	Culture
*Staphylococcus aureus*	SBS	Culture
*Salmonella* spp.	SBS	Culture
*Pseudomonas aeruginosa*	SBS	Culture
*Pasteurella pneumotropica*	EAD	PCR
CAR bacillus	SBS	Serology
*Clostridium piliforme*	SBS	Serology
*Corynebacterium kutscheri*	SBS	Culture
*Corynebacterium bovis*	EAD	PCR
*Klebsiella pneumoniae*	SBS	Culture
*Klebsiella oxytoca*	SBS	Culture
*Streptococcus pneumoniae*	SBS	Culture
Parasites		
Fur mites	EAD	PCR
*Syphacia* spp	EAD	PCR
*Aspicularis tetraptera*	EAD	PCR
*Giardia muris*	EAD	PCR
*Spironucleus muris*	EAD	PCR
*Cryptosporidium*	EAD	PCR
*Encephalitozoon cuniculi*	SBS	Serology
Fungi		
*Pneumocystis murina*	SBS	PCR

Abbreviations: CM, colony mice (sentinel animals); EAD, exhaust air duct sample; SBS, soiled bedding sample.

### 4.2 Healthspan

The cohort was split into animals used for measuring lifespan only, and those used for measures of healthspan, which included glucose tolerance tests, rotarod and tissue collection, as detailed below.

#### 4.2.1 Glucose tolerance test (GTT)

Whole‐body glucose homeostasis was as previously described (Chowdhury et al., 2017; Wu et al., 2014). Briefly, food was withdrawn starting at 0800 h on the day of the experiment, and body composition assessed using quantitative MRI (below). Six hours later (1400 h), animals were placed under a small cardboard box with the tail protruding to minimize stress, while a scalpel was used to create a small nick at approximately 1 mm from the tail tip, and fasting blood glucose was recorded using a hand‐held glucose meter (Accu‐Check Performa), which required a volume of less than 1 μL.

Animals then received an i.p. injection of 25% glucose equivalent to 2 g/kg of lean body mass, as determined previously by quantitative MRI. Blood glucose was then monitored again at 15, 30, 60, 90 and 120 min with animals placed under the cardboard box for collection at each timepoint. An additional 5 μL aliquot of whole blood was also collected using heparinised microcapillary tubes (Drummond), which was expelled into ultra‐sensitive insulin ELISA plates (Crystal Chem, 90080) that were pre‐filled with ELISA dilution buffer. GTT data were compared by summarizing the total area under the curve (AUC) of glucose curves.

#### 4.2.2 Body composition

Lean and fat body mass was recorded through quantitative MRI. Animals were weighed and then placed in a Perspex tube, which was placed inside an EchoMRI instrument which non‐invasively measures body composition over a scan time of approximately 1 min.

#### 4.2.3 Rotarod

The accelerating rotarod test was used to assess motor coordination in animals every 6 months of age. The rotating rod was placed approximately 30 cm above the cage, which is high enough to cause an avoidance of fall response, but not high enough to cause physical injury during a fall. Mice were habituated on Day 1 of the trial by being placed on the rotarod at a constant speed of 4 rpm for 60 s. The following day, mice were placed on a rotarod starting at 4 rpm and accelerated to 40 rpm over a period of 5 min. Each animal was subject to three trials, with a 30 min rest period between each trial. The average result of these three trials was used to provide a single value for each animal, as shown in Results.

#### 4.2.4 Bone

Three‐point mechanical testing of bone strength was performed as previously described (Deckard et al., 2017). Briefly, long bones were stripped of surrounding soft tissue and kept moist in PBS to prevent dehydration. Femurs were placed in a specifically designed holder to fit the length of the bone. The two holding points were set up 10 mm apart for the femur, while for the longer tibias, this length was adjusted to 11.2 mm. Three point‐bending involves applying pressure to the bones to the point of mechanical failure, with the bone sitting on two support points as the third point, the lever, pushes down. The force that is required to break each bone is recorded as well as the slope measured in force over time. The force required to break the bone is measured in grams of maximum load. The measured amount of force was deployed perpendicular to the middle of the anterior side of the diaphysis. The bones were loaded until the point of mechanical failure. The ramp amplitude was 1000 μm and ramp velocity 1 was 50 μm/s, to generate a curve with a relaxation time of 1 s.

Ramp velocity 2 and fixed relaxation time 2 were 50 μm/sand 4 s. Position and load vs time were monitored. Data output were analysed by the Mach‐1 analysis software package (Biosyntech Inc, Netherlands).

### 4.3 Survival

Animals used for measures of lifespan were not subject to assays for healthspan, as described above. In this study, animals were monitored on a weekly basis for body weight and general condition, using a scoring matrix shown below (Table 2). If animals received a score of 2 or greater, monitoring frequency was increased to daily. Animals receiving a cumulative score of 5 or higher were euthanased in line with animal ethics conditions and recorded as a death. Conditions noted for scoring included not only sudden weight loss but also sudden weight gain, which was taken as evidence of tumour growth, subsequently verified by necropsy. Otherwise, the date of death was recorded on the day that animals were found dead in their cages. Animals that had to be removed from the study due to fighting and/or fight wounds were censored from the study.

**TABLE 2:** Clinical scoring checklist for regular monitoring and assessment for animal welfare.

Activity	Score		Score
Slow moving, decreased alertness	2	Decreased activity, fearful	3
Abnormal gait, isolated,	3	Severe self‐mutilation	5
Immobile, shaking, vocalizations	5	Unresponsive when stimulated	5
Mobility/gait			
Staggering	3	Lameness	5
Tiptoe	2		
Hydration			
Dry eyes	1	Sunken eyes	3
Listless skin tenting (1 s)	4	Discharge from eyes	1
Appearance			
Decreased grooming	1	Alopecia, minor abrasion	1
Soiled/rough coat	2	Nasal/ocular discharge	3
Ulcerated skin, coat ruffled,	5	Paresis/paralysis of limbs	5
Clinical signs			
Increased respiration, effort,	2	Soft stools	2
Blood in stools	5	Peri‐anal soiling	2
Shallow/laboured respirations	3	Irregular/gasping respirations	5
Seizures	5	Body temperature <34°C	3
Body weight loss >15% overnight	5	Body temperature <35°C	2
Body weight/temp deviation >20%	5		

*Note*: A cumulative score of 5 or greater resulted in euthanasia, counted in survival data as a death.

### 4.4 NMR brain metabolomics

To measure brain metabolite levels as shown in Figure 5, tissues were subject to nuclear magnetic resonance spectroscopy (NMR). Fifteen minutes prior to sacrifice, animals received an intraperitoneal injection of [1‐^13^C] glucose (543 mg/kg, 0.3 M solution, Novachem, CLM‐420‐MPT‐PK) plus [1,2^13^C] acetate (504 mg/kg, 0.6 M solution, Novachem, CLM‐113‐PK) as described previously by Nilsen et al. (2013). Mice were then euthanased by cervical dislocation and decapitated. The brain was quickly removed, dissected out and weighed before snap freezing in liquid nitrogen.

#### 4.4.1 Methanol‐chloroform extraction for carbon spectra

Snap frozen samples were ground to a fine powder on dry ice, and their weights were recorded. All steps were performed on ice and only ice‐cold solvents were added to the frozen tissue samples. Tissues were solvent extracted by first adding 1 unit (mass) tissue, with 2 units (volume) methanol and 1 unit (volume) chloroform. Samples were left on ice for 15 min before adding half a unit (volume) of chloroform and half a unit (volume) of Milli‐Q water. Samples were vortexed well and centrifuged at 13,000 rpm for 20 min at 4°C. The upper aqueous phase was separated from the lower phase and placed into a new tube on ice. Then, 3 units (volume) methanol and 2 units (volume) of Milli‐Q water were added to the lower phase. Samples were vortexed well and centrifuged at 13,000 rpm for 20 minutes at 4°C. The upper aqueous phase was separated and added to the same tube with the first upper aqueous solution. Then again, 3 units (volume) methanol and 2 units (volume) of Milli‐Q water were added to the lower phase. Samples were vortexed well and centrifuged at 13,000 rpm for 20 min at 4°C. The upper aqueous phase was separated and added to the same tube with the first and second upper aqueous solutions. The aqueous phase was lyophilised and stored at −20°C until further analysis. The lower phase was air‐dried into a pellet and resuspended in 100 units (volume) 0.4 M sodium hydroxide (NaOH) for protein quantification using the Pierce BCA Protein Assay Kit as described in 3.2.4. The lyophilised extracts were reconstituted in 200ul of deuterium oxide (D_2_O) (Merck, 1133660100) containing 2 mM [^13^C] sodium formate (Sigma‐Aldrich, 279412‐1G) as an internal standard, and 6 mM ethylenediaminetetraacetic acid (EDTA) to chelate paramagnetic ions and remove localised field inhomogeneity.

#### 4.4.2 Determination of protein concentration

Protein concentration was determined using the Pierce bicinchoninic acid (BCA) Protein Assay Kit (Thermo‐Fisher, 23225) by following the manufacturer's protocol. Briefly, both samples and standards (0–2 mg/mL bovine serum albumin) were incubated with BCA reagents (50:1 reagent A: reagent B) at 37°C for 30 min. Absorbance was then measured on the VersaMax plate reader (Molecular Devices, USA) at 562 nm.

#### 4.4.3 NMR acquisition

Reconstituted samples were transferred into a 3 mm nuclear magnetic resonance (NMR) tube for analysis. All spectra were acquired on a Bruker AVANCE III HD 600 spectrometer (Bruker, USA) fitted with a TCI cryoprobe and refrigerated sample changer. ^1^H spectra were acquired, both with and without decoupling ^13^C using bilev composite pulse decoupling, across an effective bandwidth of 48,000 Hz during the acquisition time, on a 30 s duty cycle, while ^13^C{^1^H‐decoupled} spectra were acquired on a 4 s duty cycle using continuous WALTZ‐65 decoupling. Total metabolite pool sizes were determined using TOPSPIN v4.1 (Bruker, USA) from the ^1^H{^13^C‐decoupled} spectra and the concentration of ^13^C‐labelled compounds from ^13^C{^1^H‐decoupled} spectra, following appropriate adjustment for relaxation and nuclear Overhauser effect made according to previously acquired fully relaxed ^13^C spectra as previously described (Rowlands et al., 2017).

Animals were excluded from data analysis if they did not have complete label incorporation of ^13^C glucose or if they displayed significant increases in cerebral glutamine and reductions in myo‐inositol, indicating symptoms of sporadic congenital portosystemic shunt (Cudalbu et al., 2013).

### 4.5 Data analysis

All data were analysed and prepared for figures in the statistical programming language R, with relevant scripts used here uploaded (see data availability section). Briefly, survival data were analysed and visualised into Kaplan–Meier survival curves using the ‘*survminer’* package in R. Other data were visualised using the ggplot2 package, and analysed as indicated in figure legends using factorial ANOVAs.

## AUTHOR CONTRIBUTIONS

LEW, DLC, DAS and NT conceived of this study and obtained funding. LEW, NT designed and supervised experiments, and analysed and interpreted results. CF, NLB, MJK, HG, BPW, AD, HM, CL, LJK, ASAW, MBM, RM, SEH, AD, NAY and CDR carried out experiments and analysed results. LEW wrote and prepared this manuscript with assistance from NT.

## FUNDING INFORMATION

LEW is supported by an American Federation for Aging Research (AFAR)/Hevolution New Investigator award. This work was supported by the National Health and Medical Research Council (NHMRC) of Australia, through project grant APP1066172 and fellowship APP1127821.

## CONFLICT OF INTEREST STATEMENT

LEW and DAS are advisors and shareholders in EdenRoc Sciences, the parent company of Metro Biotech NSW and Metro Biotech which are developing NAD^+^ precursors as therapeutics, and in Life Biosciences LLC and its daughter companies. DAS is also a consultant, inventor, board member and in some cases a founder and investor in Metrobiotech, developing NAD boosters and Galilei, developing SIRT6 activators, Life Biosciences, InsideTracker, Fully Aligned, Zymo, Immetas, Animal Biosciences, Tally Health and others. For a complete list of activities, see https://genetics.med.harvard.edu/sinclair/.

## Data Availability

Annotated R scripts for data analysis and the preparation of figures have been uploaded to a Mendeley data sharing site (doi:10.17632/rdwgnfh23g.1), including relevant CSV data files.
